# Over-D1 dissection may question the value of radiotherapy as a part of an adjuvant programme in high-risk radically resected gastric cancer patients

**DOI:** 10.1038/sj.bjc.6602468

**Published:** 2005-03-15

**Authors:** M Scartozzi, E Galizia, F Graziano, V Catalano, R Berardi, A M Baldelli, E Testa, D Mari, R R Silva, S Cascinu

**Affiliations:** 1 Department of Medical Oncology, Università Politecnica delle Marche-Azienda Ospedaliera Ospedali Riuniti, Ancona, Italy; 2 Department of Medical Oncology, Ospedale di Urbino, Italy; 3 Department of Medical Oncology, Azienda Ospedaliera S Salvatore, Pesaro, Italy; 4 Department of Medical Oncology, Ospedale di Fabriano, Italy

**Keywords:** gastric cancer, lymphadenectomy, adjuvant radiochemotherapy

## Abstract

The aim of our analysis was to assess retrospectively the effect on local relapse, overall survival (OS) and disease-free survival (DFS) of a limited or an extended lymphadenectomy in radically resected gastric cancer patients. This study was performed in order to identify a subgroup of patients possibly not benefiting from a therapeutic approach such as chemoradiation therapy. We divided our patients into two groups according to lymphadenectomy type: group A for limited (<25 resected lymph nodes) and group B for extended (>25 resected lymph nodes) lymph nodes resection. A total of 418 patients were analysed: tumour stage at diagnosis was pT2–3 pN1–3 M0 in 339 patients and pT3 N0 M0 in 79 patients. Median age at diagnosis was 68 years (range 30–92 years). A total of 306 patients (73.2%) were in group A and 112 (26.8%) in group B. The median survival time (OS) for patients in groups A and B was 58.8 and 84.8 months, respectively (*P*=0.0371); median DFS was 28.8 months in group A and 59.9 months in group B (*P*=0.0027). At multivariate analysis, extension within the gastric wall, nodal involvement and the number of resected lymph nodes appeared to affect both OS and DFS. An inadequate lymph nodes resection can affect survival and result in a higher incidence of local relapse, making the latter group of patients optimal candidates for adjuvant chemoradiation.

Although in the last few years the overall incidence of gastric cancer has gradually decreased worldwide, it remains second only to that of lung cancer, with an estimated 755 000 new cases diagnosed annually around the world (Jemal *et al*, 2002). Although many advances have been made in the diagnosis and treatment of gastric cancer, the global outcome for patients diagnosed with this disease is still disappointing with a 5-year survival rate not exceeding 30% of all cases in Western Countries (Bouvier *et al*, 2002; Nitti *et al*, 2003).

In operable gastric cancer, both the extent of surgery and the value of adjuvant treatment remain matter of scientific debate, with surgery still representing the cornerstone of any curative procedure (van de Velde and Peeters, 2003; Hohenberger and Gretschel, 2003). Nevertheless, controversy still exists about the adequate extent of lymphadenectomy, which may be limited to perigastric lymph nodes (D1 dissection) or may include removal of the lymphatic chains along the celiac axis, the common hepatic and splenic artery and the hilus of the spleen (D2 dissection) (Kim and Karpeh, 2002; Alberts *et al*, 2003; Nitti *et al*, 2003).

Although a D2 dissection is the generally accepted surgical procedure in Japan, the debate about the benefits of D1 *vs* D2 lymph nodes dissection is still ongoing (Bonenkamp *et al*, 1999; Cuschieri *et al*, 1999; Davis and Sano, 2001; Lewis *et al*, 2002; Reis *et al*, 2002; Sierra *et al*, 2003).

More recently, a general agreement has been achieved about a so-called over-D1 lymphadenectomy (D1 dissection and retrieval of at least 25 nodes) on the basis of the finding that the probability of accurate assessment of lymph node status increases with the number of nodes resected, with a plateau reached at 20–25 nodes (Marubini *et al*, 2002).

Although this recommendation addresses to the principle that lymph nodes are regarded as indicators rather than governors of disease, several clinical data also indicated that a lymph nodes resection including more than 25 lymph nodes could have a major impact on the global outcome of patients who underwent apparent radical surgery for gastric cancer (Siewert *et al*, 1998; Marubini *et al*, 2002). It has been suggested that this improvement could be mainly due to a reduction in local recurrences (Kim and Karpeh, 2002).

Furthermore, it has been recently reported that a chemoradiotherapy adjuvant approach may improve the outcome of radically resected gastric cancer by lowering the incidence of local relapse (Macdonald *et al*, 2001).

The aim of our analysis was to assess retrospectively the possible effects on local relapse, and consequently on overall survival (OS) and disease-free survival (DFS), of limited or extended lymphadenectomy in a homogeneous group of radically resected gastric cancer patients. This analysis was performed in order to identify a subgroup of patients not likely to benefit from a toxic therapeutic approach such as chemoradiation therapy.

## PATIENTS AND METHODS

### Patients selection

The study population was selected from a central database that includes 765 patients with gastric cancer operated in four different institutions.

At the end of each surgical resection, all nodes were dissected from the specimen by a member of the surgical team. Classification of the N-factor was made according to the numeric system introduced by the fifth TNM (N1: metastases in 1–6 regional lymph nodes; N2: metastases in 7–15 regional lymph nodes; N3: metastases in more than 15 regional lymph nodes) (Sobin and Wittekind, 1997).

A total of 418 patients were eligible for our analysis: 339 patients with pT2–3 pN1–3 M0 (tumour invading muscolaris or serosa (T2–3), lymph node metastasis in one to more than 15 regional lymph nodes (N1–3), no distant metastasis (M0)) and 79 patients with pT3 N0 M0.

Our analysis was limited to pT3N0, pT2–3N+ tumours since they either represent the largest group of patients candidates to radical surgery and those in which an adjuvant therapy may have a role in improving prognosis.

A dissection of <25 or more than 25 lymph nodes was, respectively, classified as limited or extended lymphadenectomy. A cutoff of 25 resected lymph nodes has been arbitrarily chosen according to data reported by Marubini *et al* (2002), Siewert *et al* (1998) and Wagner *et al* (1991). Therefore, we divided our patients into two groups: group A for limited and group B for extended lymph nodes resection. All surgical centres involved presented comparable characteristics for the number of gastric cancer patients seen and the number of surgical procedures performed per year. All surgeons involved had the same scientific background and training.

Follow-up of both groups occurred at 3 months intervals for 2 years, then at 6 months intervals for 3 years and yearly thereafter. Follow-up consisted of physical examination, a complete blood count, chest radiography and CT scanning as clinically indicated. The site and date of first relapse and the date of death were recorded.

### Data management and statistical analysis

Statistical analysis was performed with SAS software version 8.2 for Windows (SAS Institute Inc., Cary, NC, USA).

The association between categorical variables was estimated by *χ*^2^ test.

Survival distribution was estimated by the Kaplan–Meier method (Kaplan and Meier, 1958). Significant differences in probability of relapsing between the strata were evaluated by log-rank test.

Cox multiple regression analysis was used to assess the role of lymphadenectomy as prognostic factor adjusted for those variables significant at univariate analysis (Cox, 1972).

Hazard ratios and 95% confidence intervals were estimated from regression coefficients. A significant level of 0.05 was chosen to assess the statistical significance.

For statistical analysis, OS and DFS were defined, respectively, as the interval between radical surgery and death or last follow-up visit and as the interval between radical surgery and clinical progression or death or last follow-up visit if not progressed.

## RESULTS

A total of 418 patients were eligible for our analysis: 339 patients with pT2–3 pN1–3 M0 and 79 patients with pT3 N0 M0. A total of 249 patients were males (59.6%) and 169 females (40.4%), and median age at diagnosis was 68 years (range 30–92). A total gastrectomy was performed in 249 patients (59.6%), whereas the remaining patients underwent a subtotal resection of the stomach.

A total of 306 patients (73.2%) received a limited and 112 (26.8%) an extended lymph nodes dissection. In all, 210 patients (50.2%) were classified as N1, 98 (23.5%) as N2, 31 (7.4%) as N3 and 79 (18.9%) as N0. In the whole group, a median of 18 (range 0–68) lymph nodes were resected. A total of 66 patients (15.8%) had a pT2 and 352 (84.2%) had a pT3 gastric cancer (Table 1).

With a median follow-up of 25 months (range 1–130 months), we recorded information on the site of the first relapse and these sites were categorised as local or distant (Table 2). Local recurrence occurred in 23% of cases with <25 lymph nodes resected and in 4.7% of those with >25 lymph nodes resected (*P*=0.0001); however, 37% of those with <25 lymph nodes resected and 24.8% of those with >25 lymph nodes resected had distant relapses (*P*=0.12).

**Figure 1:**
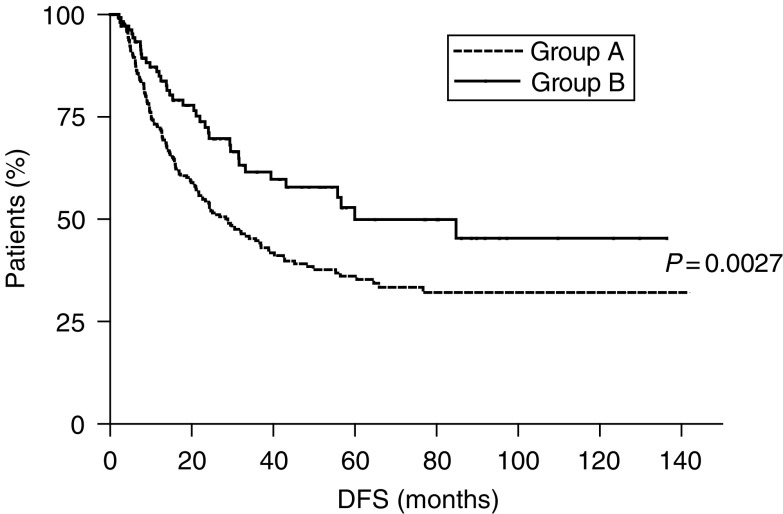
Disease-free survival of gastric cancer patients undergoing limited (⩽25 lymph nodes resected, group A - - - -) or extended (>25 lymph nodes resected, group B —) lymphadenectomy.

The median DFS for patients in groups A and B was, respectively, 28.8 and 59.9 months (*P*=0.0027) (Figure 1). At multivariate analysis, extended lymph node dissection appeared an independent prognostic factor for DFS (HR=0.52, CI 0.36–0.74, *P*=0.0003), which was also influenced by extension within the gastric wall (pT2 *vs* pT3; HR=0.41, CI 0.26–0.63, *P*=0.0001) and nodal involvement (N0 *vs* N+; HR=0.33, CI 0.21–0.52, *P*=0.0001).

**Figure 2:**
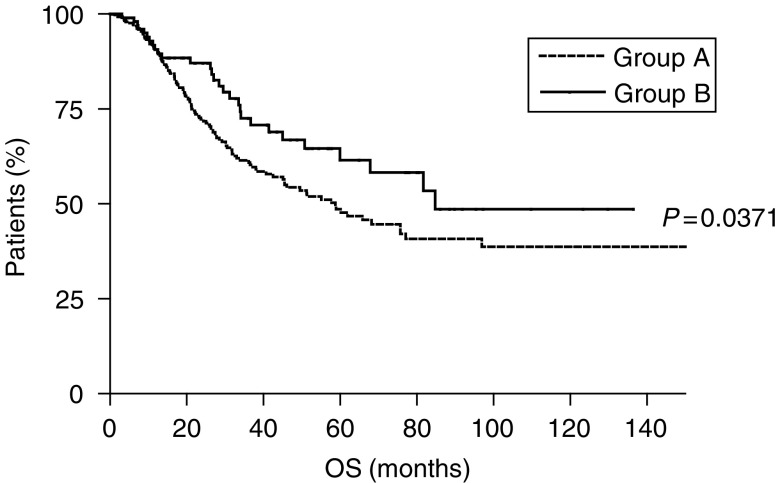
Overall survival of gastric cancer patients undergoing limited (⩽25 lymph nodes resected, group A - - - -) or extended (>25 lymph nodes resected, group B —) lymphadenectomy.

The extension of lymphadenectomy also resulted determinant in median survival time (OS) with an OS of 58.8 months for patients in group A and 84.8 months for patients in group B (*P*=0.0371) (Figure 2).

At multivariate analysis, three variables seemed to influence OS: extension within the gastric wall (pT2 *vs* pT3; HR=0.47, CI 0.29–0.76, *P*=0.002), nodal involvement (N0 *vs* N+; HR=0.46, CI 0.29–0.74, *P*=0.0015) and the number of resected lymph nodes (>25 *vs* <25; HR=0.59, CI 0.39–0.89, *P*=0.012).

These results were confirmed in subgroups analysis, separately considering N-positive and N-negative patients. In fact, among N-positive patients, OS was 51.3 months for those who received limited lymphadenectomy and 84.8 months for those who received extended lymphadenectomy (*P*=0.0316).

Also, DFS appeared to be influenced by the extension of lymphadenectomy in this latter group of patients, as it was 22.4 months for patients who underwent limited lymphadenectomy and 56.6 months for patients who underwent extended lymphadenectomy (*P*=0.0036).

**Figure 3:**
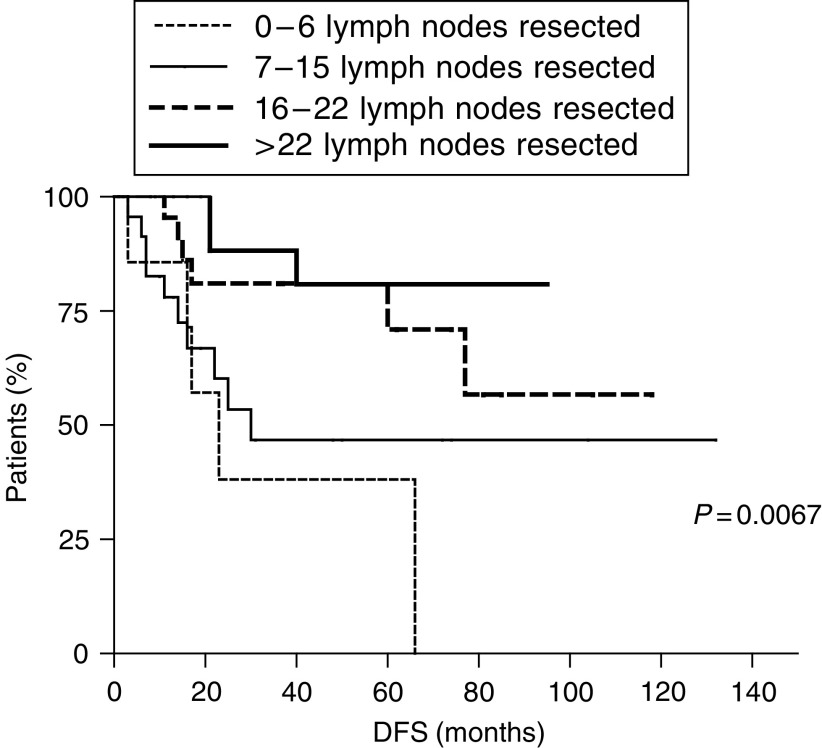
Disease-free survival in lymph nodes-negative gastric cancer patients. Kaplan–Meier curves show that a progressively more extended lymphadenectomy correlates with a progressively better DFS.

**Figure 4:**
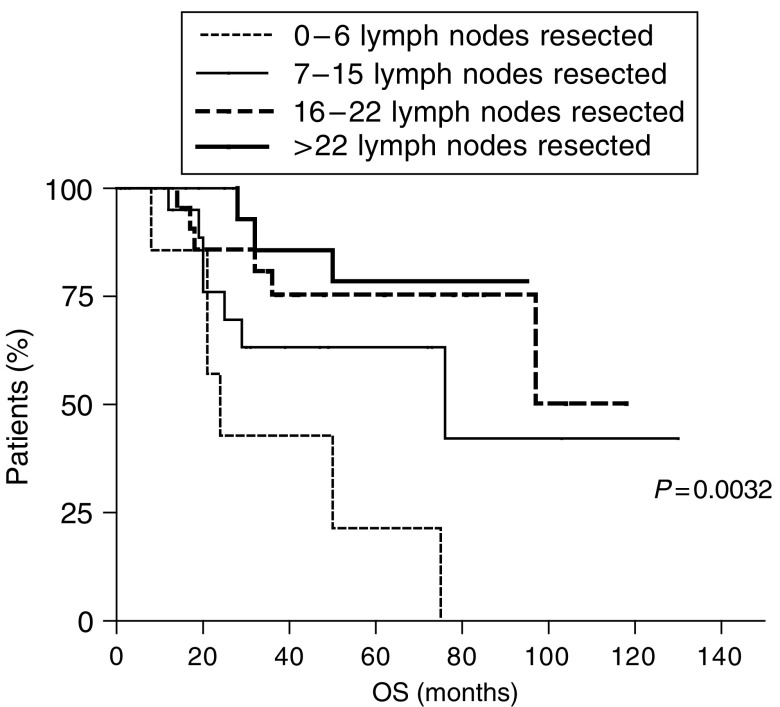
Overall survival in lymph nodes-negative gastric cancer patients. Kaplan–Meier curves show that a progressively more extended lymphadenectomy correlates with a progressively better OS.

In N-negative patients, DFS was progressively affected by a progressively more extended lymph nodes dissection: at a follow-up of 25 months (range 1–130 months), the DFS of patients with 0–6 and 7–15 resected lymph nodes was, respectively, 23 and 30 months, while it was not reached for patients with 16–22 and with more than 22 resected lymph nodes. The difference between the four survival curves was statistically significant (*P*=0.0067) (Figure 3). Similar results were observed for OS: for patients with 0–6 and 7–15 resected lymph nodes, OS was, respectively, 24 and 76 months, while OS was not reached for patients with 16–22 and with more than 22 resected lymph nodes (*P*=0.0032) (Figure 4).

## DISCUSSION

One of the main reasons for disease progression in apparently radically resected gastric cancer patients is represented by the frequent occurrence of locoregional relapse (Macdonald *et al*, 2001; Gunderson, 2002; Park *et al*, 2003). This high incidence of local relapse provided the rationale for the INT-0116 trial (Macdonald *et al*, 2001). In fact, this trial showed that a chemoradiotherapy adjuvant approach was able to improve prognosis mainly by lowering the incidence of local relapse (from 29 to 19%); on the contrary, a limited or no effect was seen on metastatic diffusion.

The quality of surgical treatment in this latter study can be considered inadequate, as a substantial proportion of patients (54%) underwent a D0 resection (no clearance of the lymphatics). On the basis of these considerations, the results of the INT-0116 trial could also be interpreted according to the hypothesis of having treated residual disease by radiochemotherapy in a significant subgroup of patients (Macdonald *et al*, 2001; Hohenberger and Gretschel, 2003).

Our results seem to confirm these conclusions. In fact, patients undergoing an optimal surgery experienced a local relapse rate as low as 4.7%, whereas this was 21% in patients receiving a suboptimal surgery. Another important aspect arising from our analysis is once again the lack of effect of optimal surgery on distant metastases. Similar findings were found in the INT-0116 study, when the effects of radiochemotherapy on distant metastases were analysed.

The results we presented in our study seem to go along with data from Siewert *et al* (1998) and Macdonald *et al* (2001) (also Hundahl *et al*, 2002). In these two studies, a survival advantage was suggested for extended lymphadenectomy and in our analysis, the median survival time for patients in groups A and B was, respectively, 58.8 and 84.8 months (*P*=0.0371). An apparent imbalance for the type of gastrectomy (i.e. total *vs* subtotal gastrectomy in the two groups) could be noticed in our series. In fact, about 84% of the patients in group A had a total gastrectomy compared to 50.7% of those in the limited lymphadenectomy group (group B). Therefore, it can be postulated that this latter factor may have affected recurrence and survival. Nevertheless, it has already been demonstrated that the extent of gastrectomy (total or subtotal) has no impact on the global outcome of radically resected gastric cancer patients (Bozzetti *et al*, 1997).

Although not demonstrated in our data analysis, which did not directly address the role of adjuvant treatment in gastric cancer patients, these findings seem to question the role of adjuvant chemoradiotherapy as a standard of practice in all gastric cancer patients, whereas its role could be that of a partially compensation for an inadequate surgery.

The question remains whether novel and effective chemotherapeutic agents could have a role along with optimal surgery, in order to further improve survival in optimally resected gastric cancer patients.

Therefore, we can hypothesise that patients undergoing suboptimal surgery and consequently at higher risk for local relapse may be suitable for postoperative radiotherapy, whereas those receiving optimal surgery may be candidates to adjuvant chemotherapy as they are considered at higher risk for metastatic progression.

We then believe that our findings could help planning future trials of postoperative management of gastric cancer patients, especially now that new drugs are to be urgently tested in this setting.

## Figures and Tables

**Table 1:** Patients characteristics

	**Whole group**	**Group A (%)**	**Group B (%)**
Number	418	306 (73.2)	112 (26.7)
Age (range)	68 (30–92)	68 (30–92)	67 (33–87)
Sex			
Male	249	189 (61.8)	60 (53.6)
Female	169	117 (38.2)	52 (46.4)
Gastrectomy			
Total	249	155 (50.7)	94 (84)
Subtotal	168	151 (49.3)	18 (16)
pT stage			
pT2	66	53 (17.3)	13 (11.6)
pT3	352	253 (82.7)	99 (88.4)
pN stage			
pN0	79	59 (19.3)	20 (17.9)
pN1	210	165 (53.9)	45 (40.2)
pN2	98	73 (23.9)	25 (22.3)
pN3	31	9 (2.9)	22 (19.6)

**Table 2:** Local and distant relapse

**Resected lymph nodes**	**Patients**	**Local relapse (%)**	**Distant relapse (%)**
⩽25	306	71 (23%)	115 (37%)
>25	112	5 (4.7%)	28 (24.8%)
*P*-value		0.0001	0.12
